# Supplementary figures and images for: Let’s be pals again: major systematic changes in Palaemonidae (Crustacea: Decapoda)
Source: PeerJ. 2015 Aug 13;3:e1167. doi: 10.7717/peerj.1167 (PMC4558070; doi:10.7717/peerj.1167)

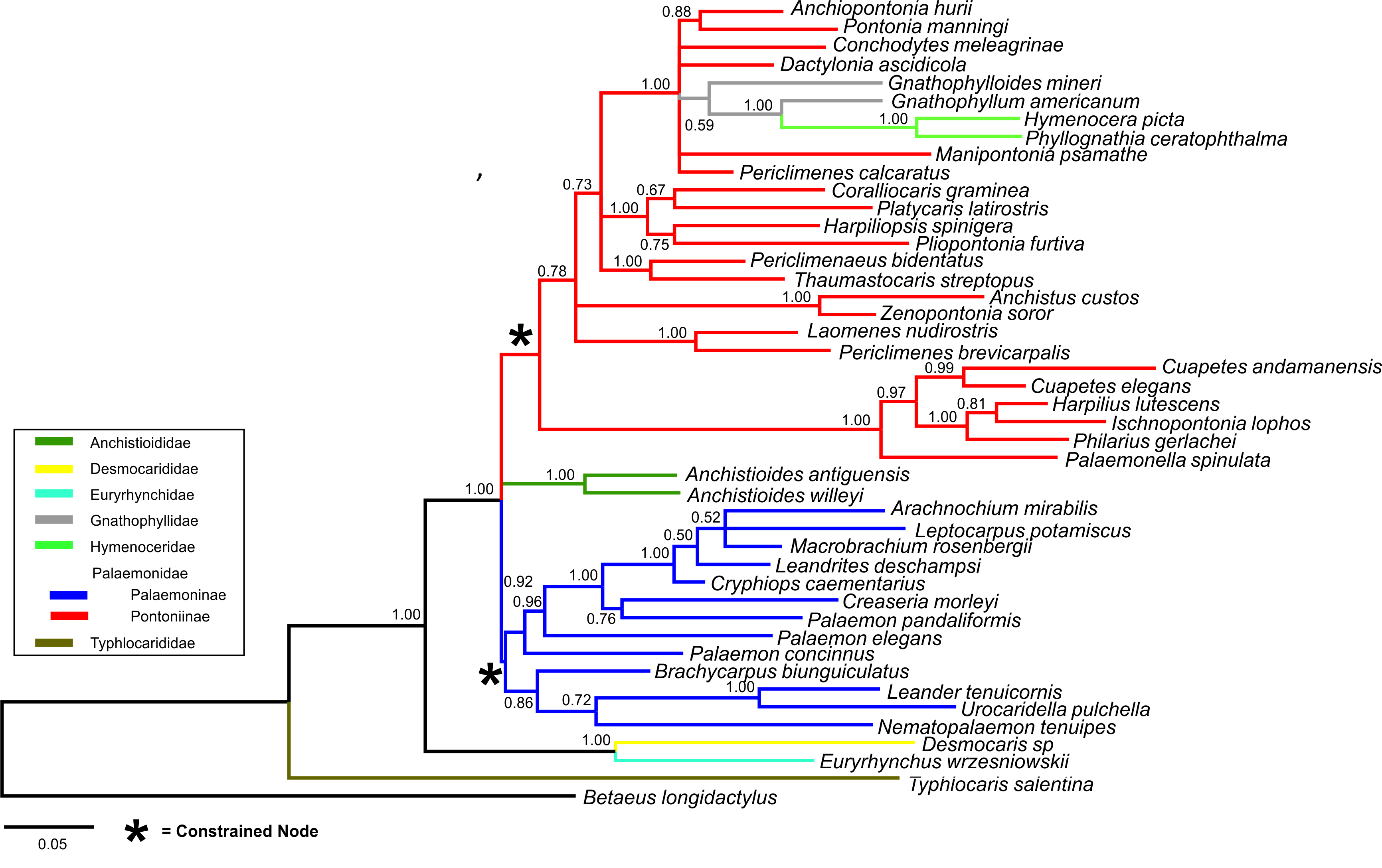

Supplement: Figure S1 — Constraint B (Palaemoninae species form a clade and species of Pontoniinae, Gnathophyllidae, and Hymenoceridae form a clade) (clades with >0.50 posterior probability shown) (Tree Score = −16546.48). [file peerj-03-1167-s001.png]

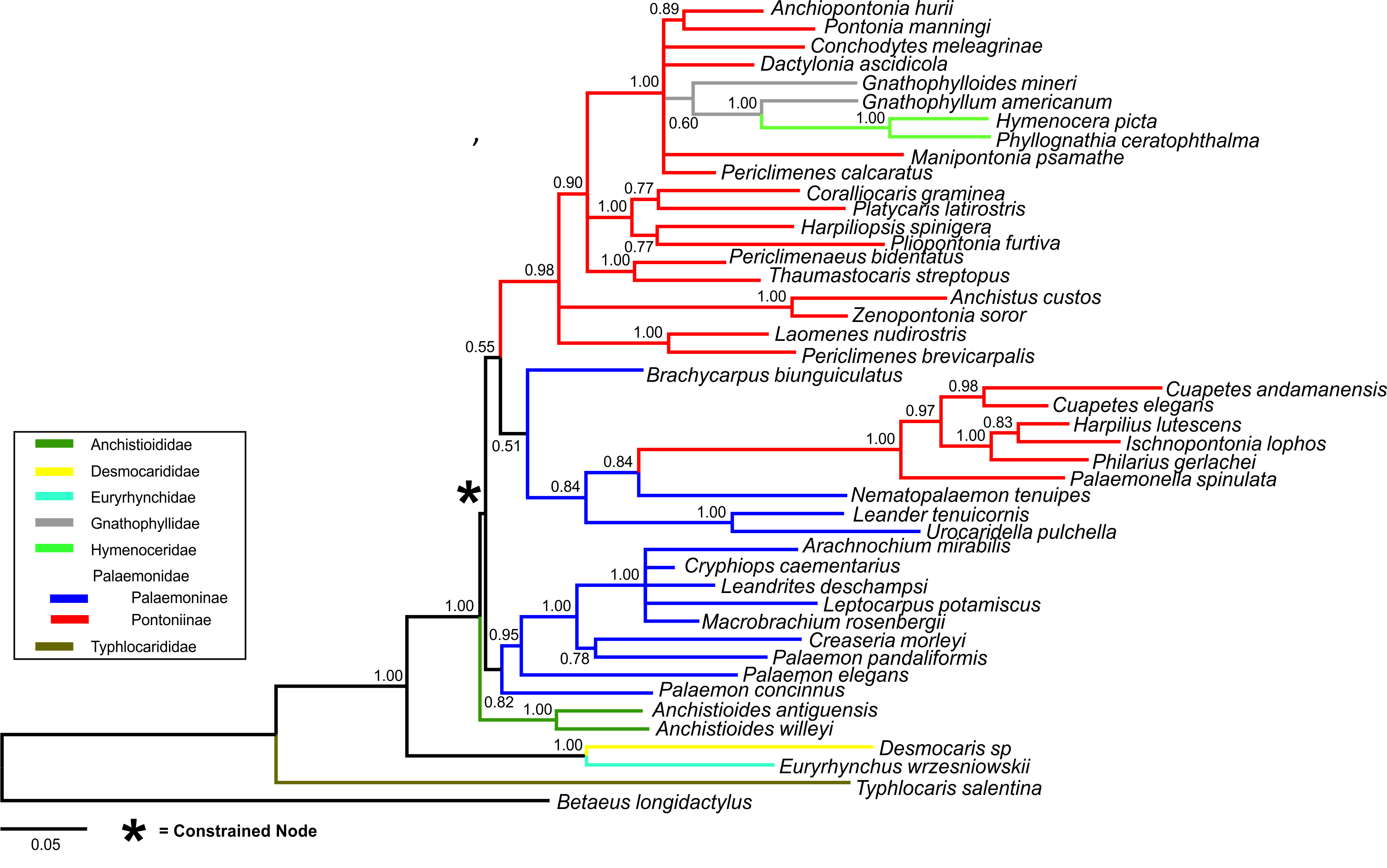

Supplement: Figure S2 — Constraint C (species of Palaemoninae, Pontoniinae, Gnathophyllidae and Hymenoceridae all form a clade) (clades with >0.50 posterior probability shown) (Tree Score = −16540.26). [file peerj-03-1167-s002.png]

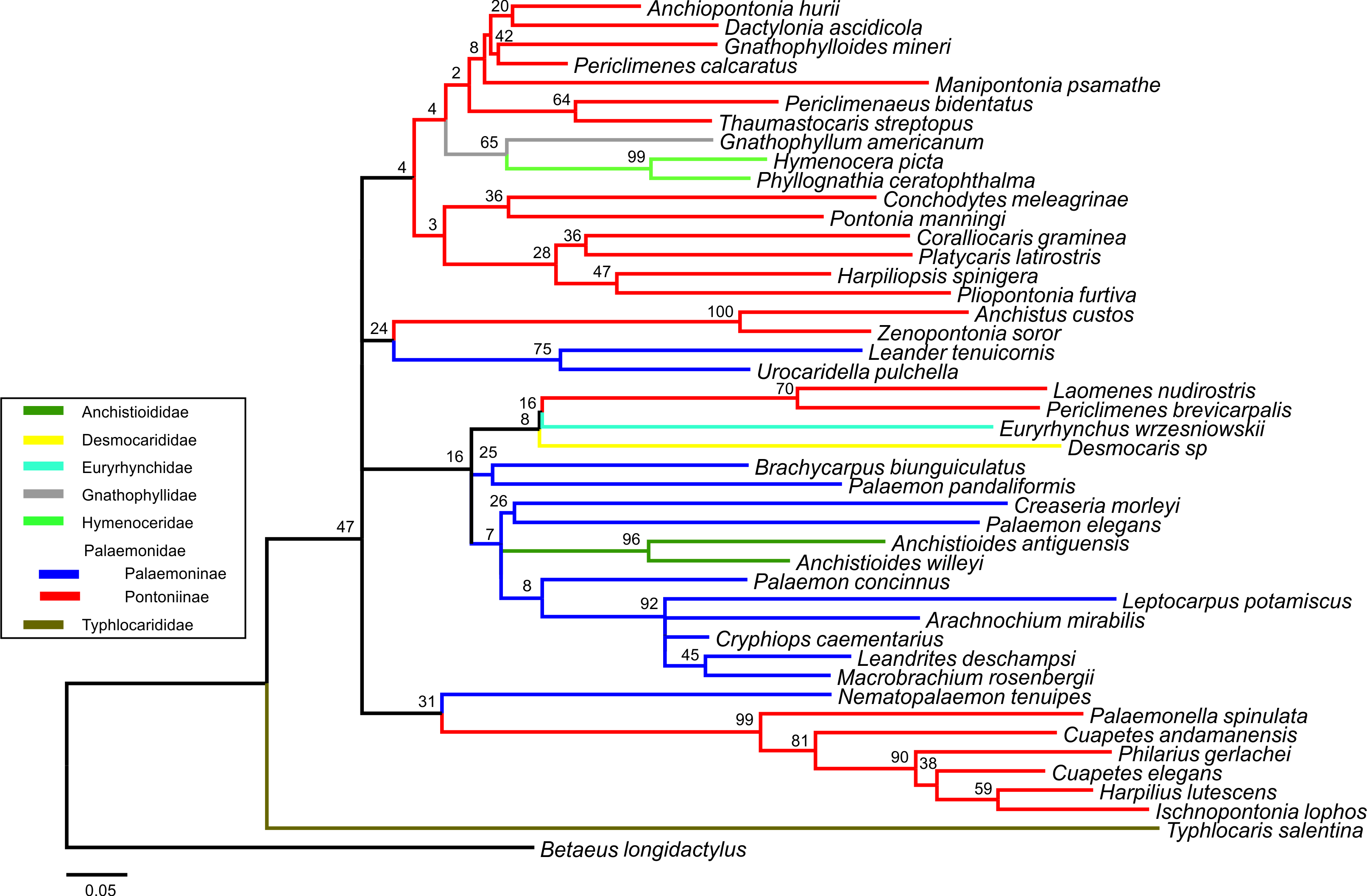

Supplement: Figure S3 [file peerj-03-1167-s003.png]

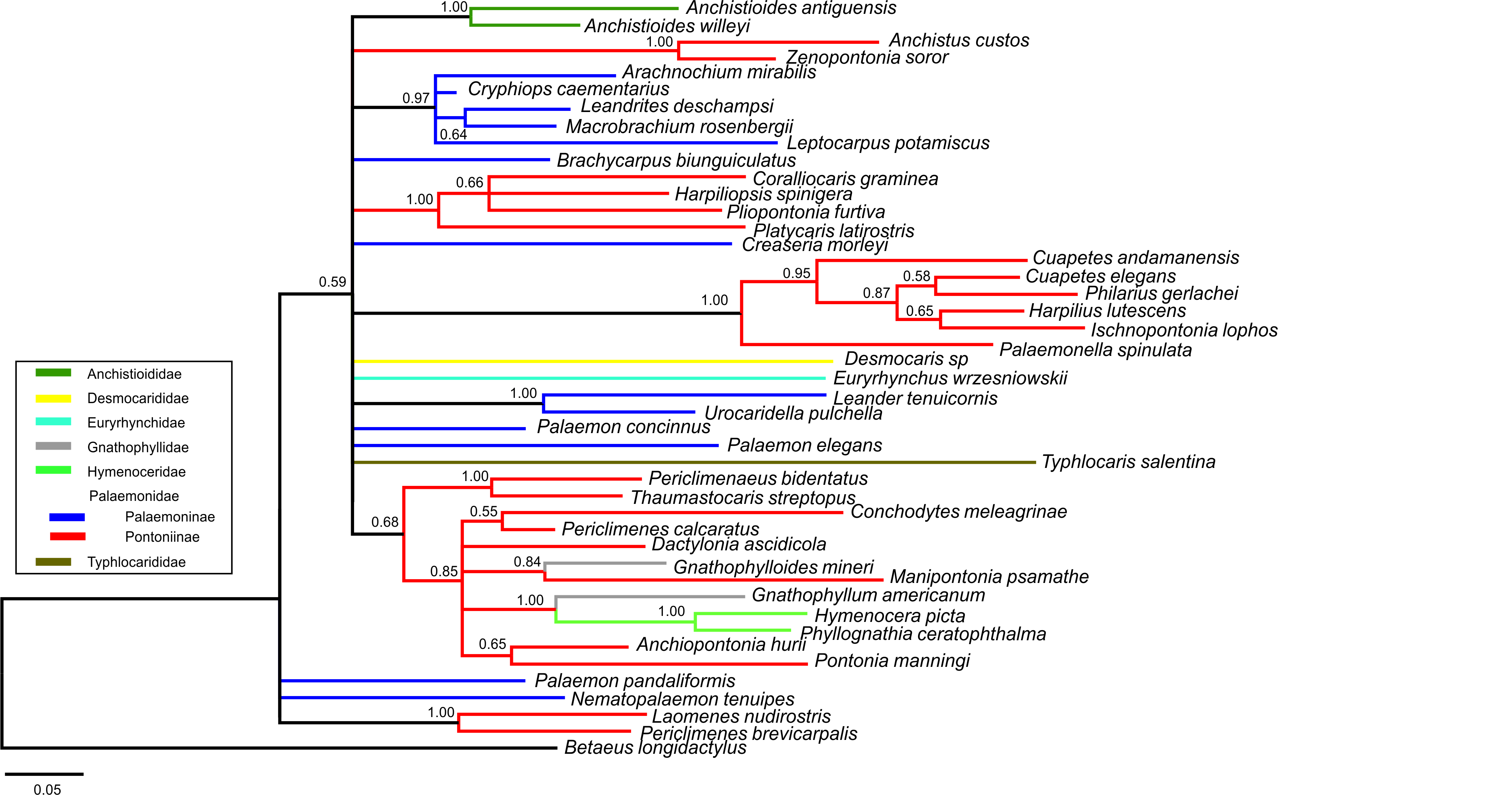

Supplement: Figure S4 [file peerj-03-1167-s004.png]

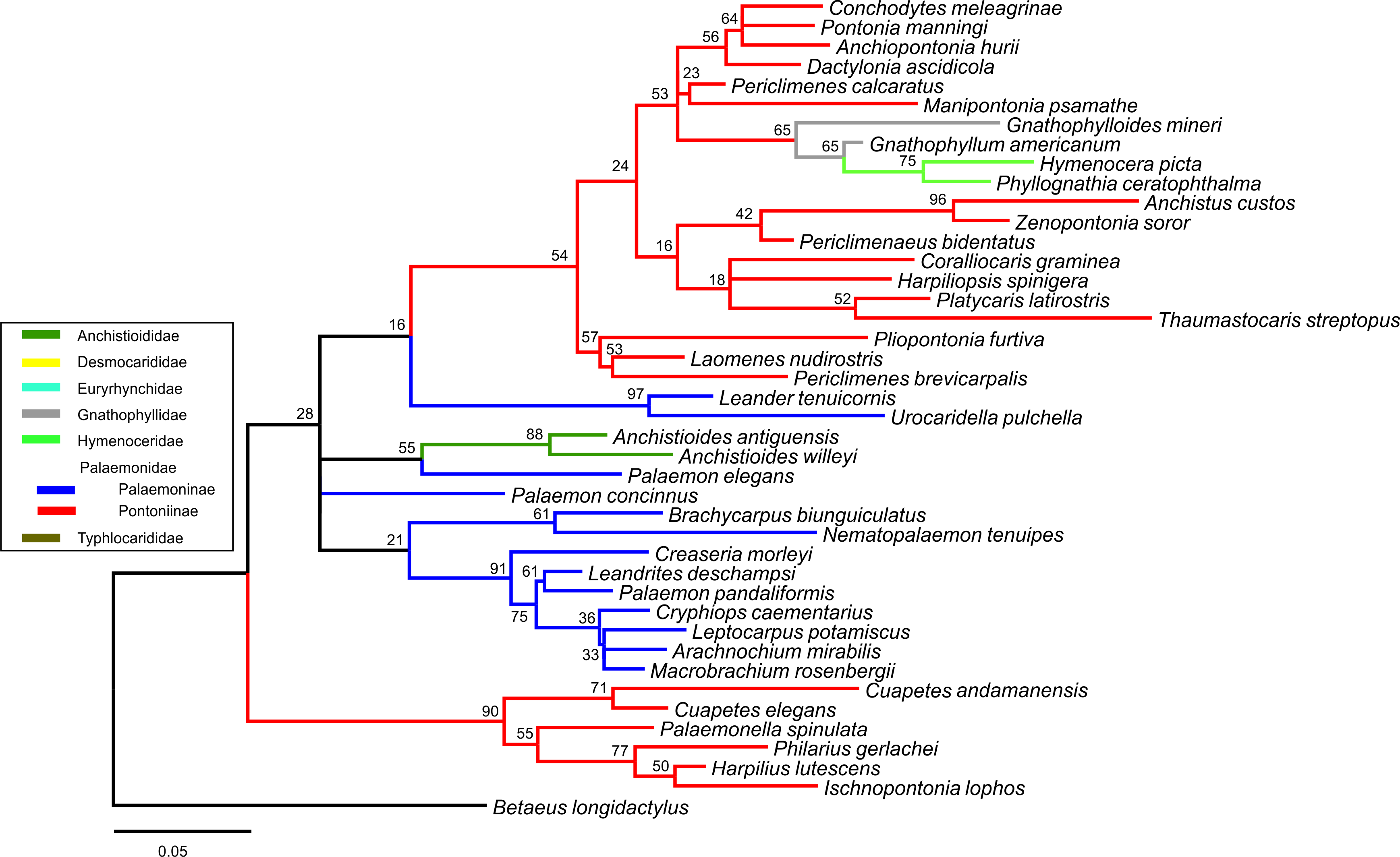

Supplement: Figure S5 [file peerj-03-1167-s005.png]

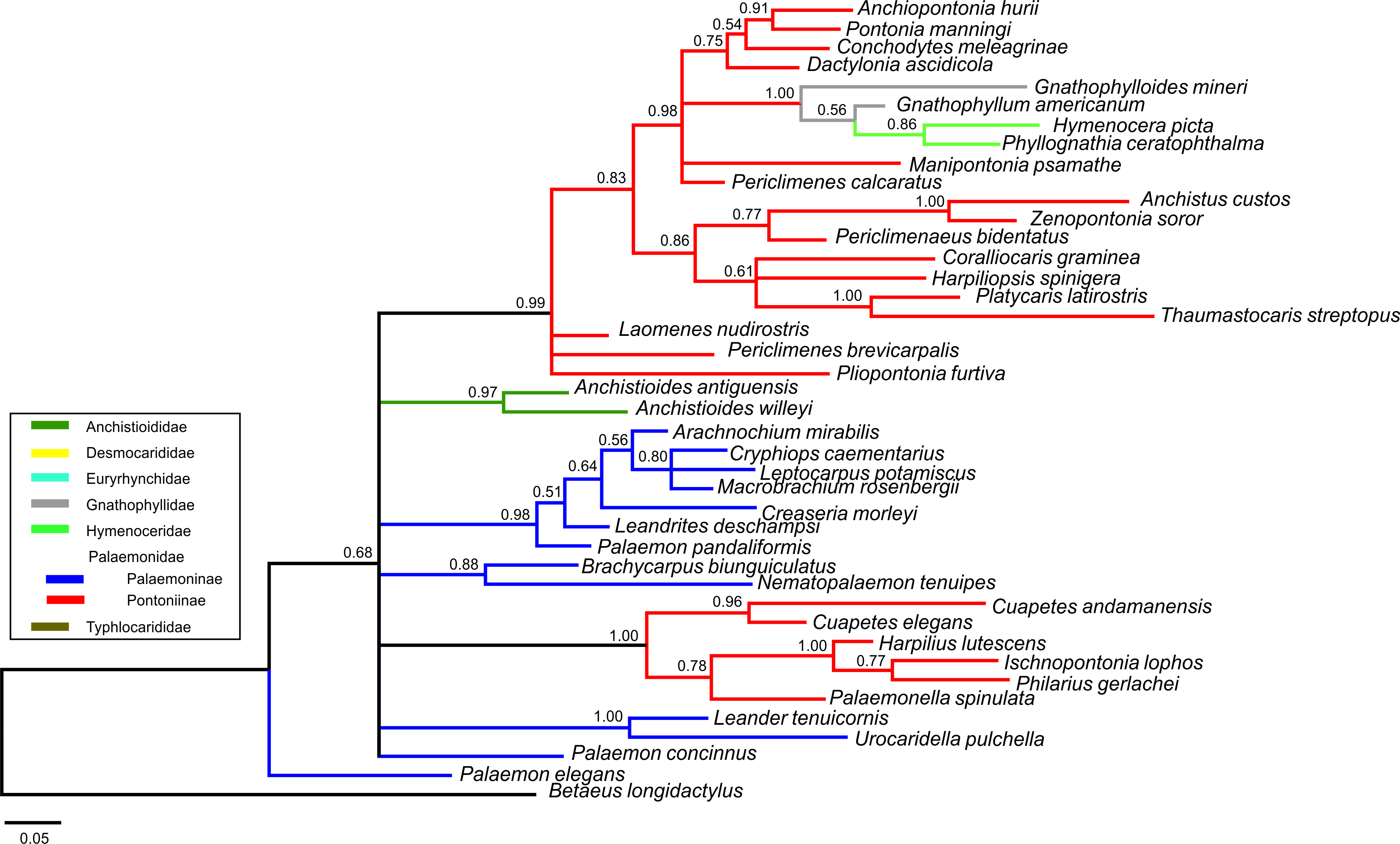

Supplement: Figure S6 [file peerj-03-1167-s006.png]

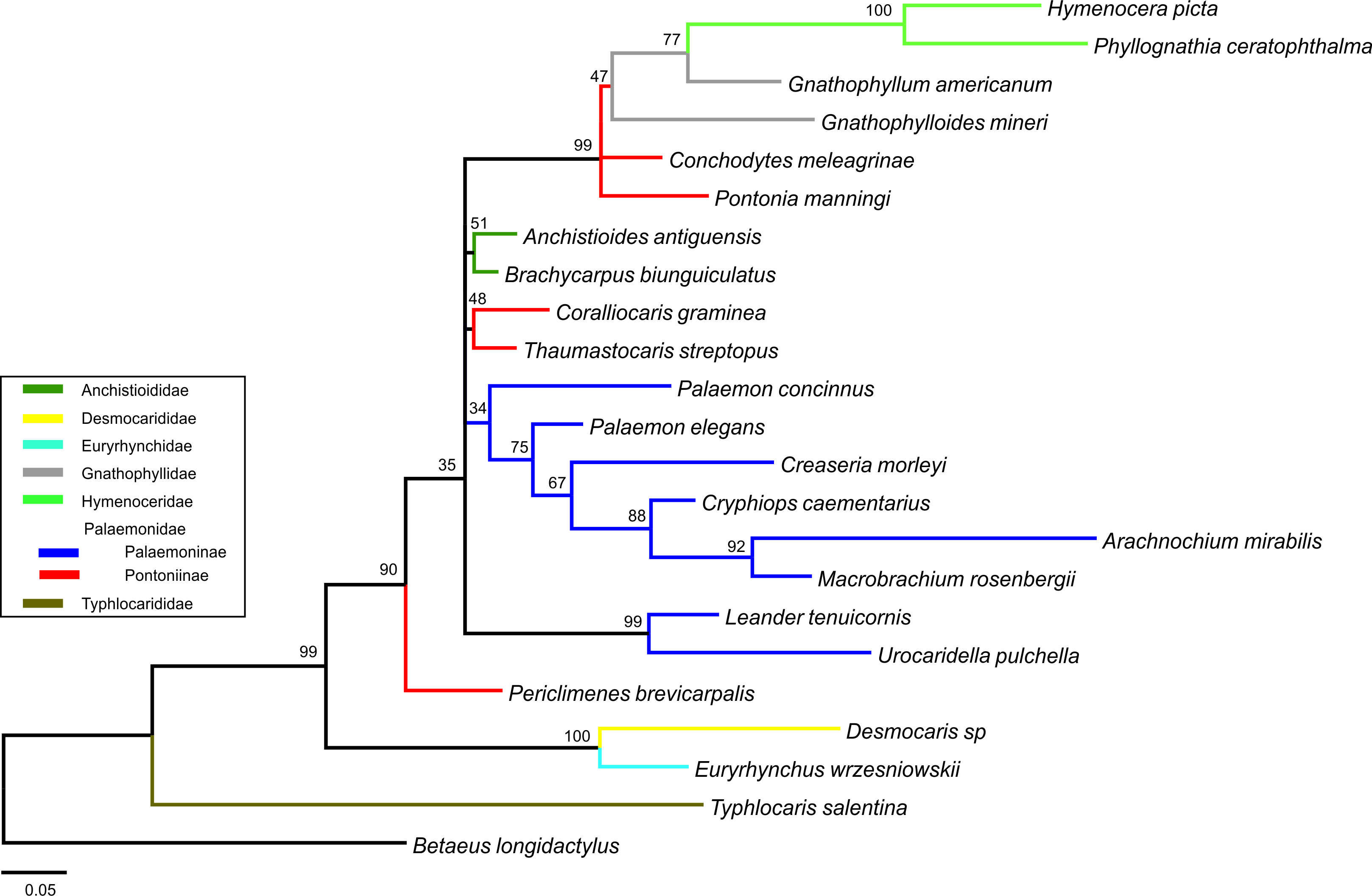

Supplement: Figure S7 [file peerj-03-1167-s007.png]

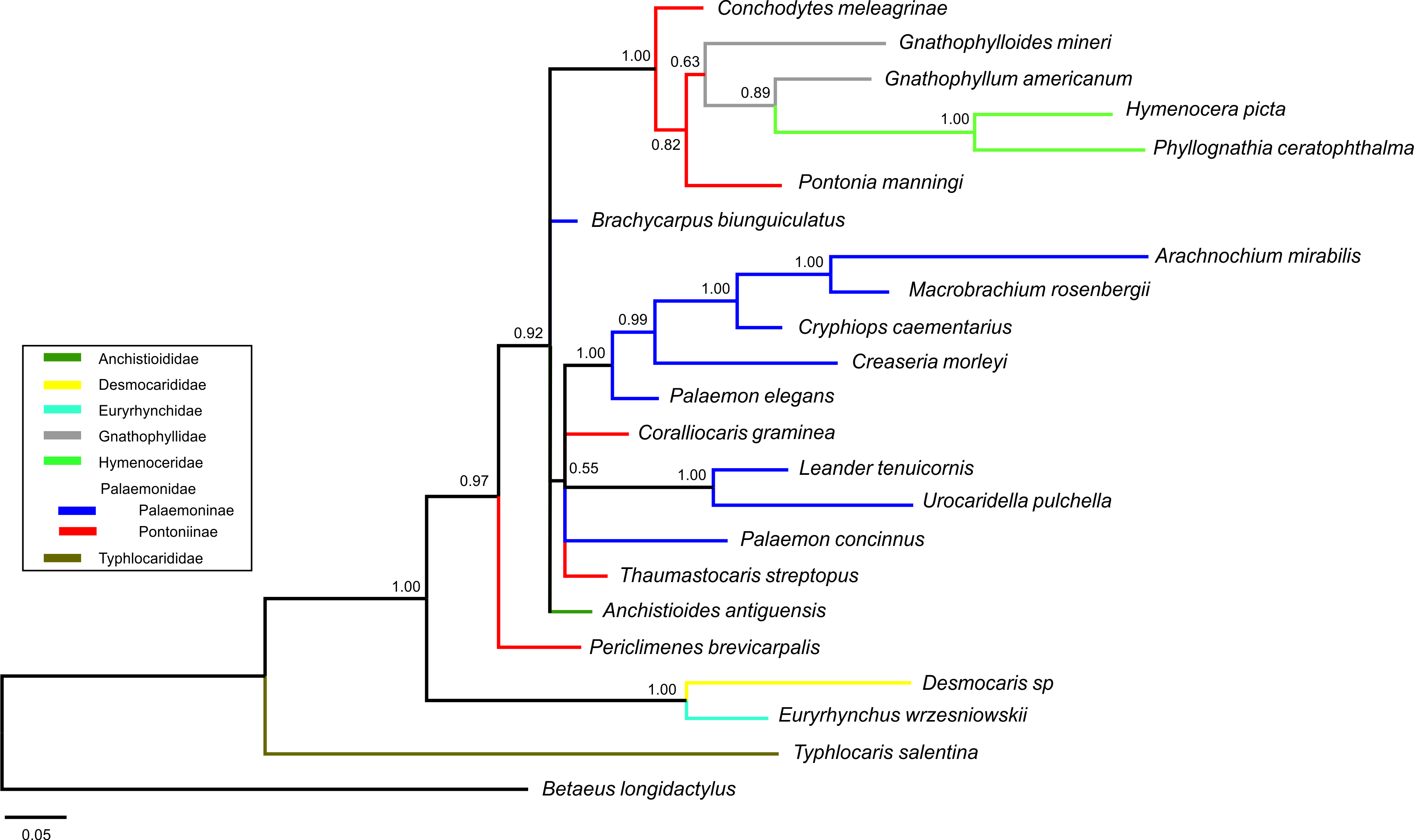

Supplement: Figure S8 [file peerj-03-1167-s008.png]
